# Supplementary material for: A Window into Mammalian Basement Membrane Development: Insights from the mTurq2-Col4a1 Mouse Model
Source: bioRxiv. 2023 Sep 27:2023.09.27.559396. Preprint. [Version 1] doi: 10.1101/2023.09.27.559396 (PMC10557719; doi:10.1101/2023.09.27.559396)
Supplement: Supplement 2 [file media-2.docx]

**Table S1 – sgRNA and oligonucleotide sequences**

| **Name** | **Sequence (5’-3’)** |
| --- | --- |
| sgRNA1 for *mTurq2-Col4a1* CRISPR* | GCGCAGCCGAGCAGCTGCGAAG |
| sgRNA2 for *mTurq2-Col4a1* CRISPR | CTGCGAAGGTGAGTTCCCTGCGGG |
| *mTurq2-Col4a1* genotyping F | CCTCCGAGACTGAGCACCTC |
| *mTurq2-Col4a1* genotyping R | TCTCGTTGGGGTCTTTGCTC |
| *mTurq2-Col4a1* genotyping R (het vs hom) | AAATCTCACTGGCTCCTCGGTA |

*sgRNA used to generate the line
