# Supplementary figures and images for: A Window into Mammalian Basement Membrane Development: Insights from the mTurq2-Col4a1 Mouse Model
Source: bioRxiv. 2023 Sep 27:2023.09.27.559396. Preprint. [Version 1] doi: 10.1101/2023.09.27.559396 (PMC10557719; doi:10.1101/2023.09.27.559396)

## Adult epidermis

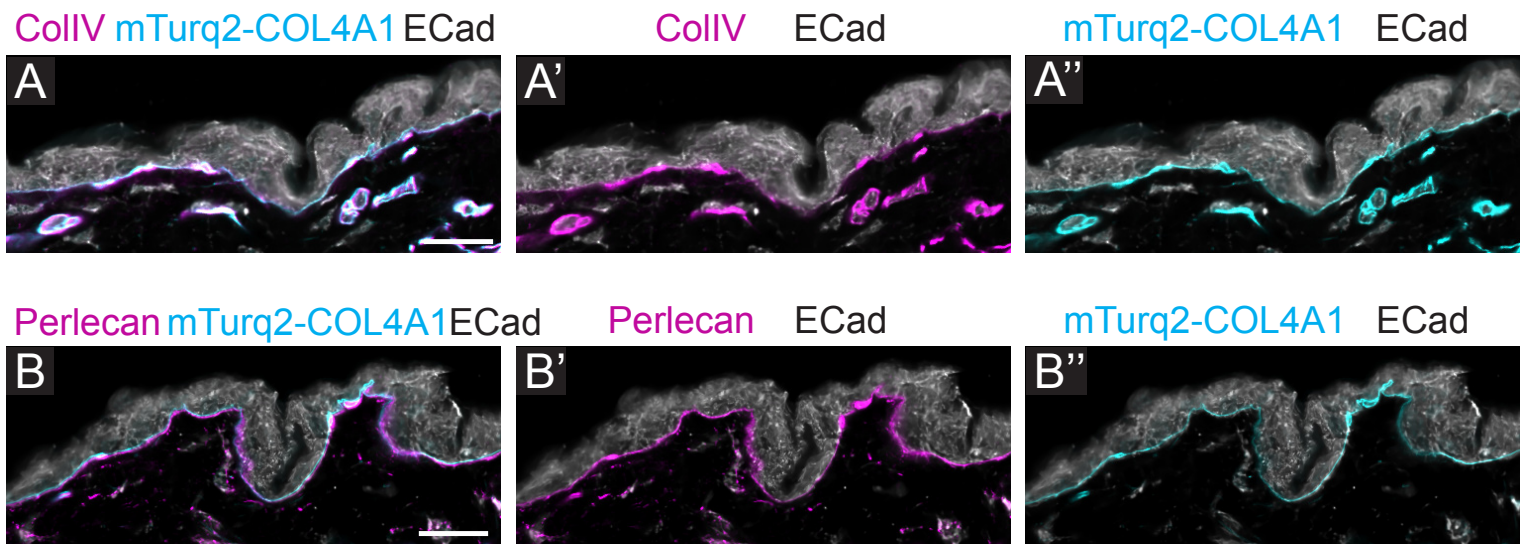

## Adult kidney

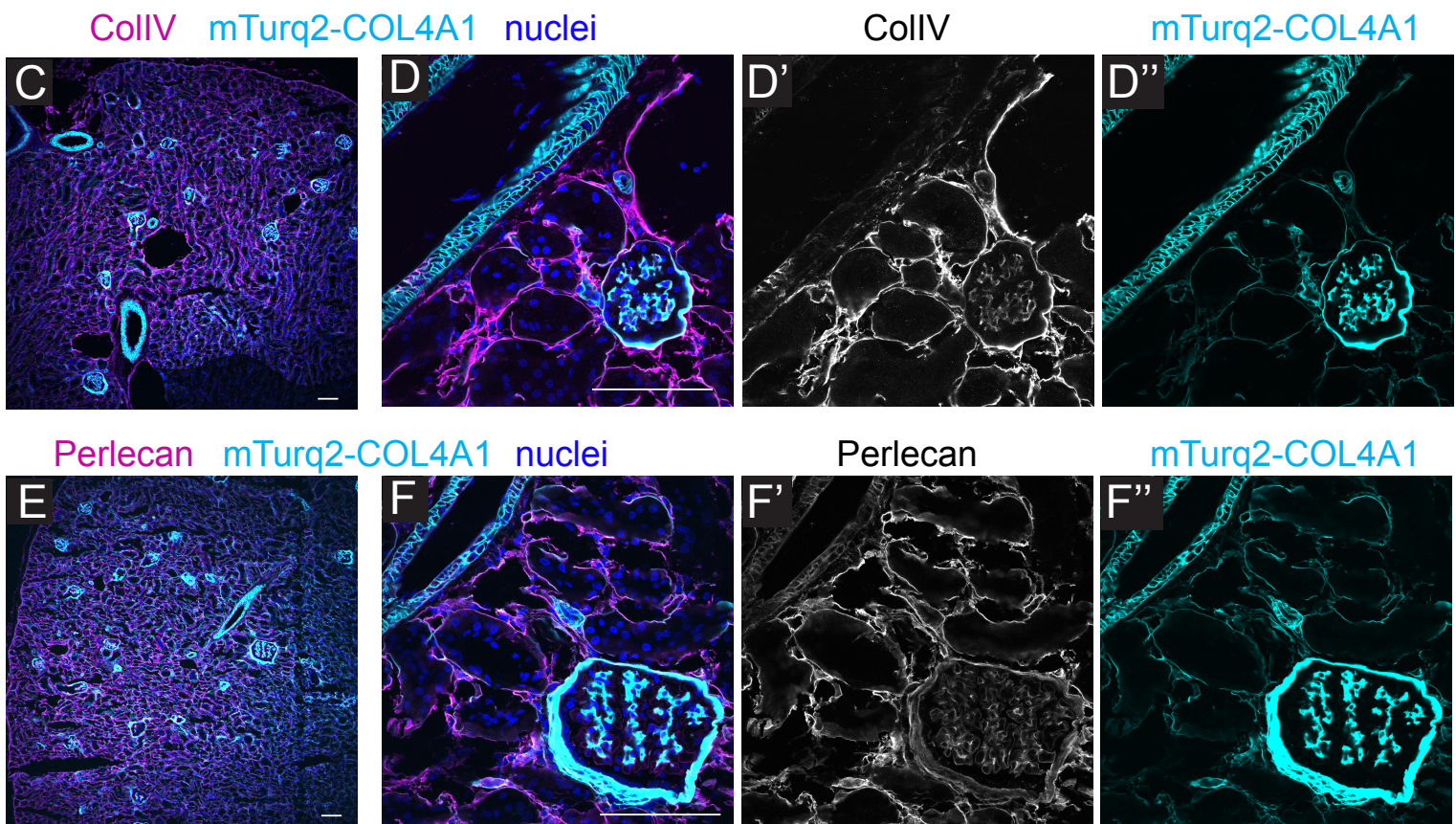

Supplement: Supplement 6 — Supplemental Figure 1, related to Figure 1. mTurq2-Col4a1 basement membrane localization in adult epidermis and kidney. (A-F) Additional representative images from a second biological replicate mTurq2-Col4a1/+ adult mouse. (A-B) Dorsal skin sections from mTurq2-Col4a1/+ adult mouse labelled with ColIV (A, A’; magenta) or Perlecan (B, B’, magenta) and E-Cadherin (grayscale). mTurq2-COL4A1 (cyan) localizes to basement membrane underlying the interfollicular epidermis and the dermal vasculature. Scale bars 20 μm. (C) Additional biological replicate of kidney sections from mTurq2-Col4a1/+ adult mouse labeled with ColIV antibodies (magenta) and Hoechst to mark nuclei (blue). Scale bar 100 μm. (D-D”) High magnification view of mTurq2-COL4A1 (cyan) localization in the kidney of second biological replicate mTurq2-Col4a1/+ adult mouse labeled with ColIV (magenta in G; grayscale in G’) Hoechst (blue). Scale bar 100 μm. (E) Kidney section from mTurq2-Col4a1/+ adult mouse labeled with Perlecan antibodies (magenta) and Hoechst (blue). Scale bar 100 μm. (F-F”) High magnification view of mTurq2-COL4A1 (cyan) localization in the kidney of mTurq2-Col4a1/+ adult mouse labeled with Perlecan (magenta in G; grayscale in G’) Hoechst (blue). Scale bar 100 μm. [file media-6.pdf]

Supplemental Figure 3

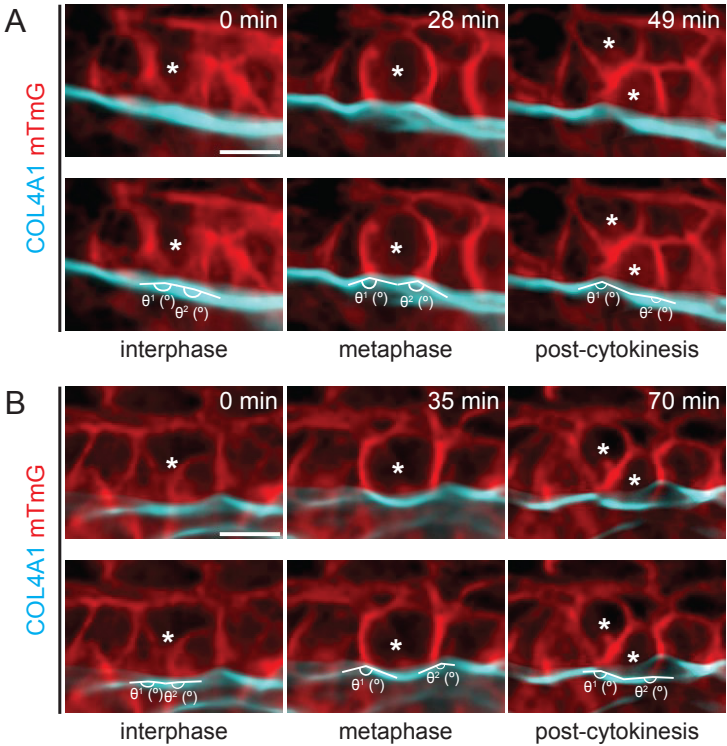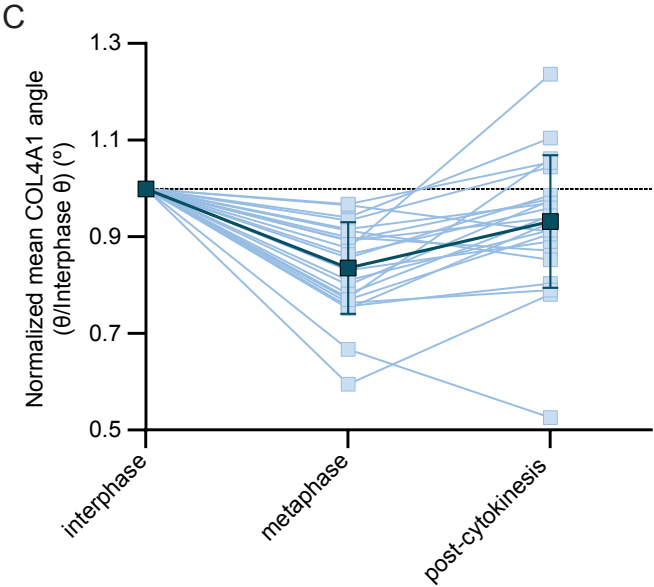

Supplement: Supplement 7 — Supplemental Figure 3, related to Figure 5. The epidermal BM deforms around dividing basal progenitor cells. (A) Additional example of snapshots taken from time-lapse live imaging movie of basal cell division in E15.5 mTurq2-Col4a1/+;mTmG/mTmG backskin. Interphase, metaphase, and post-cytokinesis stages are shown (left to right). Bottom panel shows same images with membrane curvature measurements shown. Scale bar 10 μm. (B) Additional example as (A) (C) Quantification of the average angle of BM deformation as shown in Figure 5F. Data is normalized to the interphase angle for each individual dividing cell. Each line represents one dividing cell. Dark blue line represents average of all dividing cell lines. n=23 dividing cells. Error bars = mean+SD. [file media-7.pdf]

E18.5

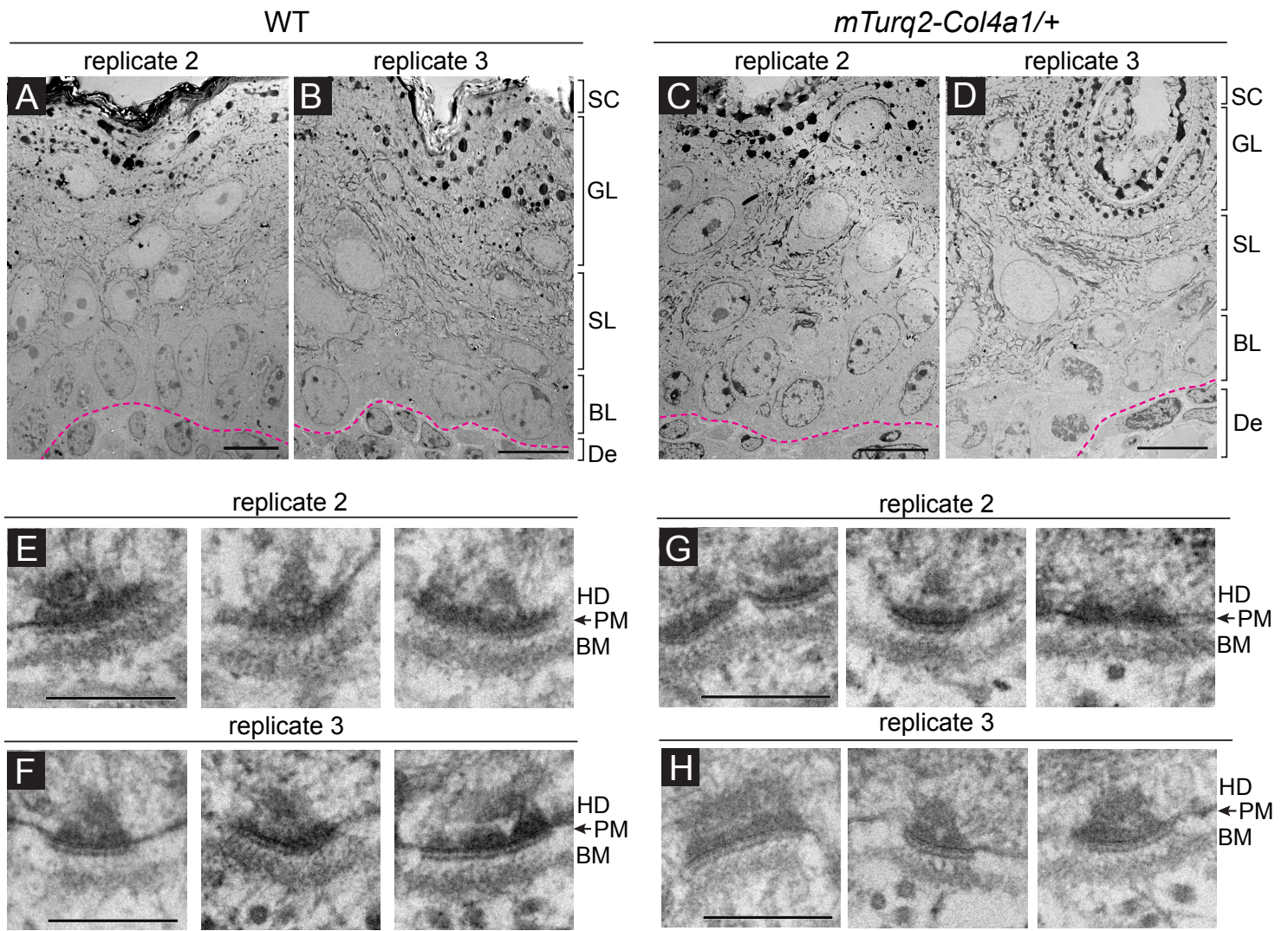

Supplement: Supplement 8 — Supplemental Figure 2, related to Figure 3. Ultrastructural organization of the epidermis and dermal-epidermal junction in mTurq2-Col4a1/+ embryonic skin. (A-D) Representative TEM images from additional experimental replicates of E18.5 WT control (A-B) and mTurq2-Col4a1/+ (C-D) embryonic skin. Der = dermis, BL = basal layer, SL = spinous layer, GL = granular layer, SC = stratum corneum. Dotted line denotes dermal-epidermal boundary. Scale bars 10 μm. (E-H) Representative images of individual hemidesmosomes at the dermal-epidermal junction region of WT control (E-F) and mTurq2-Col4a1/+ (G-H) embryonic skin. Hd = hemidesmosome, PM = plasma membrane, BM = basement membrane. Scale bars 250 nm. [file media-8.pdf]
